# Lifetime Induced Abortion: A Comparison between Women Living and Not Living with HIV

**DOI:** 10.1371/journal.pone.0095570

**Published:** 2014-04-21

**Authors:** Flávia Bulegon Pilecco, Luciana Barcellos Teixeira, Álvaro Vigo, Michael E. Dewey, Daniela Riva Knauth

**Affiliations:** 1 Graduate Studies Program in Epidemiology, Federal University of Rio Grande do Sul (Universidade Federal do Rio Grande do Sul), Porto Alegre, Rio Grande do Sul, Brazil; 2 Graduate Studies Program in Public Health, Federal University of Rio Grande do Sul (Universidade Federal do Rio Grande do Sul), Porto Alegre, Rio Grande do Sul, Brazil; 3 Department of Professional Assistance and Guidance, Nursing School, Federal University of Rio Grande do Sul (Universidade Federal do Rio Grande do Sul), Porto Alegre, Rio Grande do Sul, Brazil; 4 Department of Statistics, Federal University of Rio Grande do Sul (Universidade Federal do Rio Grande do Sul), Porto Alegre, Rio Grande do Sul, Brazil; 5 Department of Health Service and Population Research, Institute of Psychiatry, King's College London, London, United Kingdom; 6 Department of Social Medicine, Federal University of Rio Grande do Sul (Universidade Federal do Rio Grande do Sul), Porto Alegre, Rio Grande do Sul, Brazil; University of New South Wales, Australia

## Abstract

**Background:**

Studies aimed at understanding the association between induced abortion and HIV are scarce and differ on the direction of the association. This paper aims to show the prevalence of induced abortion in a sample of pregnancies of women living and not living with HIV/Aids, determining variables associated with pregnancy termination and linked to the life course of women and to the specific context of the pregnancy.

**Methods:**

Data came from a cross-sectional study, using interviewer-administered questionnaire, developed with women that attended public health services in Porto Alegre, Brazil. A generalized estimating equation model with logit link measured the association between determinants and abortion.

**Findings:**

The final sample was composed of 684 women living with HIV/Aids (2,039 pregnancies) and 639 women not living with HIV/Aids (1,539 pregnancies). The prevalence of induced abortion among pregnancies in women living with HIV/Aids was 6.5%, while in women not living with HIV/Aids was 2.9%. Among women living with HIV/Aids, the following were associated with induced abortion in the multivariable analysis: being older, having a higher education level, having had more sexual partners (i.e., variables linked to the life course of women), having had children prior to the index pregnancy and living with a sexual partner during pregnancy (i.e., variables linked to the context of each pregnancy). On the other hand, among women not living with HIV/Aids, only having a higher education level and having had more sexual partners (i.e., determinants linked to the life course of women) were associated with voluntary pregnancy termination in multivariable analysis.

**Conclusion:**

Although determinants are similar between women living and not living with HIV/Aids, prevalence of induced abortion is higher among pregnancies in women living with HIV/Aids, pointing to their greater social vulnerability and to the need for public policy to address prevention and treatment of HIV associated with reproductive issues.

## Introduction

Of the 210 million pregnancies that occurred worldwide in 2008, 16% resulted in unplanned births and 21% in induced abortion [1], [2].

In Brazil, as in most Latin American countries, induced abortion is classified as a crime against life, being allowed only in cases of rape, risk to the woman's life or an anencephalic fetus [3], [4]. Although illegal, abortion is widely practiced in unsafe conditions in Brazil [5]. In 2005, indirect techniques based on hospitalizations due to abortion estimated that there were 1,054,242 abortions in Brazil. In that year, the rate was 2.07 abortions per 100 women aged between 15 and 49, and the estimated number of abortions accounted for 30% of live births [6]. Data from the National Survey of Abortion, a population-based survey with 2,002 women aged between 18 and 39, conducted with the ballot box method in state capitals and metropolitan regions of Brazil in 2010 showed that 15% of surveyed women had already had at least one abortion during their lifetime [7].

Currently Brazil has about one-third of the total number of cases of HIV/Aids in Latin America (around 530,000 cases) [8], with an estimated prevalence of 0.42% (0.31% among women and 0.52% among men) [9]. In Brazil, most HIV cases still occur in men, even though the sex ratio has been slowly decreasing. In 2011, the incidence rate among men was 25.9 and among women, 14.7 cases per 100,000 inhabitants, with a sex ratio of 1.7 [10]. However, few studies show what influences reproductive decisions throughout life for these women.

Globally, studies aiming to understand the relation between induced abortion and HIV are scarce and differ on the direction of the association. A study conducted in Vietnam, involving the last pregnancy before and the first pregnancy after HIV diagnosis indicated an increase in induced abortion occurrence after diagnosis [11]. However, a study conducted in Canada, analyzing pregnancies in women living with HIV/Aids (WLHA) conceived 20 weeks before HIV diagnosis and comparing them with pregnancies conceived 20 weeks after the diagnosis, pointed in the opposite direction [12]. Moreover, a study that compared Scottish women who were intravenous drug users or had intravenous drug user partners showed no difference in induced abortion between WLHA and women not living with HIV/Aids (WNLHA) [13], while a study conducted in Australia, which retrieved the medical records of all pregnancies occurred in WLHA, found evidence indicating a higher recurrence of voluntary termination of pregnancy in WLHA when compared to women in the general population [14]. The largest survey conducted in Brazil, which aimed to compare the prevalence of induced abortions in WLHA and WNLHA, held in 13 Brazilian cities, showed a higher prevalence of abortion in WLHA, although the association between HIV and induced abortion was reduced in the multivariable analysis, after the number of partners during lifetime was included in the multivariable model [15].

The following factors are frequently associated with induced abortion among women in general: age [16]–[20], skin color (non-white) [19], [21], higher education levels, unemployment or unstable employment situation, low socioeconomic status [20], [22], having had previous pregnancies and previous children [16], [20], [21], [23], [24], being single [16], [19]–[21], [25]–[29] or in an unstable relationship [20], having a greater number of sexual partners [21] and a history of illicit drug use [30], [31] and being conducive with abortion practices [21].

The literature indicates that the determinants of induced abortion among WLHA are fundamentally the same as those among women in the general population [15], [32], [33]. Other specific factors, such as having a partner living with HIV or with an unknown serology [33], having been diagnosed before the introduction of PMTCT [11], [34] and in cases in which women perceive themselves as having poor health [35] are also determinants of induced abortion practice. In this sense, studies conducted in Brazil showed that the decision to abort is influenced by both the diagnosis of HIV and other aspects of the WLHA's life, such as the period of life in which pregnancy happened, stability and quality of relationship with the sexual partner, insertion in the labor market, and family support [36], [37].

Furthermore, some studies have analyzed factors associated with induced abortion in WLHA considering induced abortion and HIV as constant variables (i.e., even if a woman had multiple pregnancies, if she reported having abortion in just one of them, she was classified as having had abortions) [32], [34], [36]. In comparison, other studies have focused on specific pregnancies, analyzing abortion and HIV status in each pregnancy (e.g., last pregnancy before and first pregnancy after HIV diagnosis [11], [12], or pregnancies that occurred during the study period [13], [14], [35], [38]).

In this context, this paper aims to show the prevalence of induced abortion in all pregnancies of a WLHA group (both before and after HIV diagnosis) and a WNLHA group that attended public health services in Porto Alegre, Brazil, determining variables associated with pregnancy termination and linked both to the life course of women and to the specific context of the pregnancy.

## Methods

### Ethics statement

The research that led to this paper was approved by the Research Ethics Committee of the Federal University of Rio Grande do Sul (UFRGS), under N. 2008216, as well as by the ethics committees of other institutions involved, as follows: Hospital Conceição (under N. 10-011100517), Hospital de Clínicas de Porto Alegre (HCPA) (under N. 100517), and the Ethics Committee of Porto Alegre (under N. 001.049442.09.0). All interviewees signed a written informed consent term and were advised that they could refuse to participate in the survey at any time.

### Sample description and survey procedures

Data analyzed here came from the Sexual and Reproductive Health of Women in the Context of HIV/Aids in Porto Alegre Survey, a cross-sectional study conducted from January to November 2011 in public health services in Porto Alegre, Brazil. The study population was comprised of women aged between 18 and 49, who had a scheduled visit to a health professional. Survey participants answered a comprehensive set of questions on socioeconomic and demographic characteristics as well as sexual and reproductive health outcomes, regarding sexual partners, contraception, condom use, pregnancy, abortion, HIV diagnosis, and the occurrence of violence.

Participants were selected from two groups: WLHA and WNLHA. WLHA were accessed within all health services specialized in the treatment of people living with HIV/Aids, which had a specific agenda for this purpose (totaling seven health services). WNLHA were selected in primary health services. Of 159 primary health care services in Porto Alegre, 27 were selected, covering all the sanitary districts in order to obtain a representative sample of people that use public health services in the city as a whole. The number of women recruited in each health service was proportional to the number of people attending the corresponding service and interviewees were randomly selected from the daily appointment schedules of each service.

Sample size was calculated based on the prevalence of induced abortion in WLHA and WNLHA found by Barbosa et al. (2009) [15], which is the most complete study available on abortion among WLHA in Brazil to date. Considering a power of 80%, a level of significance of 5%, a sample design effect (*deff*) of 1.6 and adding 20% for possible losses, the final sample size was calculated to be 615 women in each group. Among WLHA, 65 refused to participate in the survey, as did 41 WNLHA. In the final sample, there were 684 WLHA and 639 WNLHA, with 2,039 pregnancies among WLHA (both before and after diagnosis), and 1,539 pregnancies among WNLHA.

For this analysis, the sample was restricted to women who had at least one pregnancy in their lifetime (59 WLHA and 141 WNLHA were excluded for this reason). Ongoing pregnancies (103 among WLHA and 297 among WNLHA) were excluded because their outcome was unknown at the time of the survey. After exclusions, the analyzed set consisted of 625 WLHA, who had 1,935 pregnancies, and 498 WNLHA, who had 1,242 pregnancies.

Regarding recruitment, participants were approached by interviewers (undergraduate students of nursing and nutrition and graduates in psychology, law and biomedicine) extensively trained to work with sensitive topics. These interviewers invited selected people to participate in the study, explaining procedures and applying the term of consent. Interviews were administered face-to-face by interviewers and typed directly into netbooks.

### Variables

Outcome variable was induced abortion, evaluated through a direct question, that is, if the pregnancy was terminated in a live child, a miscarriage or an induced abortion. This question was asked for each pregnancy that the women reported as having. For purposes of analysis, “live child” and “miscarriage” were combined in a category, which indicated that the pregnancy was not terminated in abortion.

The analysis used here was based on the theoretical framework proposed by Bajos et al. (2006), which states that there are characteristics common to all pregnancies linked to the life course of women, and characteristics that differ according to the specific context of each pregnancy [39]. In this paper, characteristics linked to the life course of women (surveyed at the time of the interview) included variables age, level of education, religion, race/skin color, relatives' support and number of sexual partners during lifetime. On the other hand, variables linked to the specific context of each pregnancy (surveyed in relation to the time of each pregnancy) addressed more proximal variables, such as age at pregnancy, use of contraception immediately before pregnancy, children born alive before index pregnancy and living with a sexual partner during pregnancy.

In the present paper, race/skin color was evaluated according to the Brazilian Institute of Geography and Statistics (IBGE) classification: white, black, brown (mixed), yellow (of Asian origin), and indigenous. For the purpose of analysis, race/skin color was defined as a dichotomous variable assuming (white or non-white). The question regarding relatives' support was asked for women who declared having “a relative that lives nearby”. Women who declared not having a relative that lived nearby were considered as not having relatives' support. Since previous studies present different ways of categorizing the number of sexual partners, it was chosen that this variable would be categorized in two groups, with the median as cut-off point. Regarding age at the index pregnancy, categories of age “between 30 and 39” and “between 40 and 49 were” collapsed in one due to the small number of pregnancies that occurred in the latter group. Finally, the variable children born alive was evaluated based on the information if there was a pregnancy before the pregnancy index. As to this last variable, pregnancies that were terminated in abortion (spontaneous or induced) or stillbirths were considered as not having generated a live child.

### Statistical analysis

Analysis were performed using the Stata software (StataCorp, version 11) and SAS/STAT version 9.3.

The prevalence of abortion was estimated in two ways, as follows: considering the number of women that reported having an abortion divided by the total number of women; and number of pregnancies that resulted in an induced abortion divided by the total number of pregnancies.

Variables were presented as absolute values and percentages at the descriptive analysis (Tables 1, 2, 3, and 4). In Tables 1 and 3, women were the denominator of the analysis. Differences between groups were measured by the chi-square test, calculated using the software STATA version 11. Pregnancies were the denominator of the analysis in Tables 2 and 4. In these Tables, the PROC SURVEYFREQ procedure (SAS version 9.3) was used to calculate differences between groups given the ability of this procedure to consider observations as a cluster. Moreover, the association between determinants and induced abortions was analyzed using the Rao-Scott chi-square test, a design-adjusted test that is computed by applying a design correction to the weighted Pearson chi-square statistic [40].

**Table 1:** Description of women living with HIV/AIDS and women not living with HIV/AIDS according to characteristics linked to the life course of women (variables refer to the time of the interview).

		Women living with HIV/Aids (n = 625^a^)	Women not living with HIV/Aids (n = 498^a^)	p^b^
		n (%)	n (%)	
Age at interview (years)	18–29	121 (19.4)	223 (44.8)	<0.001
	30–39	283 (45.3)	152 (30.5)	
	40–49	221 (35.3)	123 (24.7)	
Level of education (years)	0 to 7	272 (43.5)	164 (32.9)	0.001
	8 to 11	162 (25.9)	166 (33.3)	
	12 or more	191 (30.6)	168 (33.8)	
Practice of a religion	No	271 (43.4)	242 (48.6)	0.175
	Sometimes	205 (32.8)	155 (31.1)	
	Always	149 (23.8)	101 (20.3)	
Race/Skin color	White	361 (57.8)	315 (63.6)	0.046
	Non white	264 (42.2)	264 (36.4)	
Family support	Yes	339 (54.2)	309 (62.2)	0.008
	No	286 (45.8)	188 (37.8)	
Number of sexual partners in lifetime^c^	1–4	253 (44.8)	308 (66.5)	<0.001
	5 or more	312 (55.2)	155 (33.5)	

a Totals can differ due to missing answers.

b Differences between women living with HIV/Aids and women not living with HIV/Aids were calculated using chi square test.

c Categories based on median number of sexual partner at the time of the interview.

**Table 2:** Description of pregnancies occurred among women living with HIV/AIDS and women not living with HIV/AIDS according to characteristics linked to the specific context of each pregnancy.

		Pregnancies among women living with HIV/Aids (n = 1,936^a^)	Pregnancies among women not living with HIV/Aids (n = 1,242^a^)	p^b^
		n (%)	n (%)	
Age at pregnancy (years)	Up to19	582 (30.1)	399 (32.1)	0.345
	20–29	999 (51.7)	639 (51.5)	
	30–49	352 (18.2)	204 (16.4)	
Contraceptive use	Yes	817 (42.2)	451 (36.3)	0.028
	No	1,119 (57.8)	790 (63.7)	
Previous children	No	643 (33.2)	500 (40.3)	<0.001
	Yes	1,292 (66.8)	742 (59.7)	
Living with a sexual partner	Yes	1,440 (74.4)	988 (79.6)	0.004
	No	496 (25.6)	253 (20.4)	

a Totals can differ due to missing answers.

b Differences between pregnancies among women living with HIV/Aids and women not living with HIV/Aids were calculated using Rao-Scott chi-square (PROC SURVEYFREQ).

**Table 3:** Description of women living with HIV/AIDS and women not living with HIV/AIDS, with and without declaration of induced abortion, according to characteristics linked to the life course of women (variables refer to the time of the interview).

		Women living with HIV/Aids (n = 623^a^)		p^b^	Women not living with HIV/Aids (n = 493^a^)		p^b^
		Women who declared having had induced abortion (n = 81)	Women who declared having not had induced abortion (n = 542)		Women who declared having had induced abortion (n = 24)	Women who declared having not had induced abortion (n = 469)	
		n (%)	n (%)		n (%)	n (%)	
Age at interview (years)	18–29	5 (6.2)	115 (21.2)	0.001	6 (25.0)	213 (45.4)	0.125
	30–39	35 (43.2)	248 (45.8)		9 (37.5)	143 (30.5)	
	40–49	41 (50.6)	179 (33.0)		9 (37.5)	113 (24.1)	
Level of education (years)	0 to 7	33 (40.7)	239 (44.1)	0.005	2 (8.3)	162 (34.5)	0.010
	8 to 11	12 (14.8)	149 (27.5)		8 (33.3)	154 (32.8)	
	12 or more	36 (44.4)	154 (28.4)		14 (58.4)	153 (32.6)	
Practice of a religion	No	38 (46.9)	232 (42.8)	0.748	11 (45.8)	227 (48.4)	0.217
	Sometimes	24 (29.6)	181 (33.4)		5 (20.8)	150 (32.0)	
	Always	19 (23.5)	129 (23.8)		8 (33.4)	92 (19.6)	
Race/Skin color	White	52 (64.2)	309 (57.0)	0.222	16 (66.7)	297 (63.7)	0.771
	Non white	29 (35.8)	233 (43.0)		8 (33.3)	169 (36.3)	
Family support	Yes	41 (50.6)	296 (54.6)	0.501	12 (50.0)	295 (63.0)	0.199
	No	40 (49.4)	246 (45.4)		12 (50.0)	173 (37.0)	
Number of sexual partners in lifetime^c^	1–4	20 (29.0)	231 (46.8)	0.005	7 (33.3)	297 (68.0)	0.001
	5 or more	49 (71.0)	263 (53.2)		14 (66.7)	140 (32.0)	

a Totals can differ due to missing answers.

b Differences between women living with HIV/Aids and women not living with HIV/Aids were calculated using chi square test.

c Categories based on median number of sexual partner at the time of the interview.

**Table 4:** Description of pregnancies occurred among women living with HIV/AIDS and women not living with HIV/AIDS that resulted or not in induced abortion, according to characteristics linked to the specific context of each pregnancy.

		Pregnancies among women living with HIV/Aids (n = 1,935^a^)		p^b^	Pregnancies among women not living with HIV/Aids (n = 1,241^a^)		p^b^
		Ended in induced abortion (n = 126)	Not ended in induced abortion (n = 1,809)		Ended in induced abortion (n = 36)	Not ended in induced abortion (n = 1,205)	
		n (%)	n (%)		n (%)	n (%)	
Age at pregnancy (years)	Up to19	27 (21.6)	555 (30.7)	0.031	11 (30.6)	388 (32.2)	0.896
	20–29	79 (63.2)	920 (50.9)		18 (50.0)	620 (51.4)	
	30–49	19 (15.2)	332 (18.4)		7 (19.4)	197 (16.4)	
Contraceptive use	Yes	48 (38.1)	769 (42.5)	0.479	8 (22.2)	443 (36.8)	0.097
	No	78 (61.9)	1,040 (57.5)		28 (77.8)	761 (63.2)	
Previous children	No	28 (22.2)	615 (34.0)	0.026	13 (36.1)	486 (40.3)	0.635
	Yes	98 (77.8)	1,193 (66.0)		23 (63.9)	719 (59.7)	
Living with a sexual partner	Yes	52 (41.3)	1,387 (76.7)	<0.001	19 (52.8)	968 (80.4)	<0.001
	No	74 (58.7)	422 (23.3)		17 (47.2)	236 (19.6)	

a Totals can differ due to missing answers.

b Differences between pregnancies among women living with HIV/Aids and women not living with HIV/Aids calculated using Rao-Scott chi-square (PROC SURVEYFREQ).

A GEE model with a binomial response and a logit link (performed on STATA version 11) was used to determine variables independently associated with induced abortion between WLHA and WNLHA, considering all the pregnancies that these women had during life (Table 5). The GEE model is often used for the study of correlated data (assuming that observational units are grouped), particularly with binary responses [41]. In the present study, pregnancies are grouped in women. Thus, pregnancies have their own characteristics (linked to the specific context of each pregnancy) and characteristics that they share with other pregnancies (linked to the life course of women). Variables associated with induced abortion practice with a Wald test with p<0.2 in a univariable model were included in the multivariable model. Variables significant at 5% were maintained in the multivariable model. Results were expressed as odds ratios (OR), with 95% of confidence interval (CI). In order to control age difference between groups, the variable “age at interview” was kept in the multivariable model for WLHA and WNLHA.

**Table 5:** Odds ratio and 95% confidence interval for declaration of induced abortion among women living with HIV/Aids and women not living with HIV/Aids, estimated through the univariable and multivariable GEE logistic model.

		Declaration of induced abortion			
		Women living with HIV/Aids		Women not living with HIV/Aids	
		Univariable analysis OR (CI95%)^a^	Multivariable analysis OR (CI95%)^b^	Univariable analysis OR (CI95%)^a^	Multivariable analysis OR (CI95%)^b^
Age at interview (years)^c^	18–29	1	1	1	1
	30–39	2.50 (0.91–6.86)	2.30 (0.77–6.86)	1.97 (0.67–5.81)	2.76 (0.74–10.26)
	40–49	3.84 (1.41–10.47)	3.44 (1.18–10.04)	1.82 (0.63–5.27)	3.20 (0.96–10.70)
Level of education (years)^c^	0 to 7	1	1	1	1
	8 to 11	0.92 (0.45–1.87)	1.26 (0.59–2.69)	9.72 (1.99–47.55)	8.96 (1.85–43.35)
	12 or more	2.86 (1.69–4.69)	3.29 (1.84–5.88)	14.67 (3.36–64.03)	8.69 (1.91–39.33)
Practice of a religion^c^	No	1	-	1	-
	Sometimes	0.88 (0.50–1.57)	-	1.02 (0.35–2.99)	-
	Always	1.07 (0.58–1.96)	-	2.29 (0.86–6.11)	-
Race/Skin color^c^	White	1	-	1	-
	Non-white	0.61 (0.36–1.01)	-	0.94 (0.37–2.39)	-
Family support^c^	Yes	1	-	1	-
	No	0.98 (0.60–1.59)	-	1.40 (0.60–3.29)	-
Number of sexual partners in lifetime^c^ ^d^	1–4	1	1	1	1
	5 or more	2.80 (1.59–4.95)	2.34 (1.33–4.14)	4.50 (1.72–11.79)	3.85 (1.44–10.30)
Age at pregnancy (years)^e^	Up to 19	1	-	1	-
	20–29	1.37 (0.82–2.27)	-	0.92 (0.37–2.26)	-
	30–49	1.26 (0.69–2.31)	-	0.81 (0.20–3.35)	-
Contraceptive use^e^	Yes	1	-	1	-
	No	0.85 (0.50–1.43)	-	1.69 (0.62–4.61)	-
Previous children^e^	No	1	1	1	-
	Yes	2.11 (1.39–3.18)	3.42 (2.10–5.57)	1.31 (0.61–2.84)	-
Living with a sexual partner^e^	Yes	1	1	1	-
	No	3.57 (2.24–5.68)	5.00 (3.35–7.47)	3.14 (1.01–9.80)	-

a OR: Odds Ratio; CI95%: Confidence Interval of 95%. Estimated through unadjusted Generalized Estimating Equations (GEE).

b OR: Odds Ratio; CI95%: Confidence Interval of 95%. Estimated through adjusted Generalized Estimating Equations (GEE).

c Variables refer to the time of the interview.

d Categories based on median number of sexual partner at the time of the interview.

e Variables refer to the time of pregnancy.

In addition to the single model for WLHA, which contained all pregnancies that occurred during their lifetime, a regression model was done separately for pregnancies that occurred before and after the HIV diagnosis (data not shown). Regarding the model that included pregnancies occurred before the HIV diagnosis, the determinants associated with induced abortion were the same as in the model that included pregnancies both before and after diagnosis. On the other hand, the model that considered only pregnancies that occurred after HIV diagnosis showed small changes in the estimates in comparison with the model that included pregnancies occurred before and after HIV diagnosis. However, these associations were not significant because of the small number of pregnancies that ended in induced abortion after the HIV diagnosis. This lack of power to perform the analysis of serological status in each pregnancy is due to the sample size used in this study, that was calculated to analyze the lifetime prevalence of induced abortion. For this reason, when it is referred herein that a determinant is associated with induced abortion among WLHA, this abortions could have occurred both before and/or after diagnosis. The idea is to demonstrate the risk factors for abortion in this population during lifetime, and this methodological option is especially supported by the idea that determinants before and after diagnosis are comparable [34].

## Results

### Description of the sample

Among WLHA at the moment of the interview, 13.0% had had at least one abortion in their lifetime (81 of 623 WLHA declared having had a pregnancy termination). On the other hand, of 495 WNLHA, 24 (4.9%) declared having had at least one induced abortion. Of the 1,241 pregnancies that occurred in WNLHA 2.9% (36 pregnancies) ended in induced abortion. Moreover, between the 1,935 pregnancies WLHA declared having had, 126 resulted in an induced abortion (a lifetime prevalence of abortion of 6.5%). Considering pregnancy outcome and HIV diagnosis, 103 of 1,331 pregnancies that occurred before HIV diagnosis ended in an induced abortion (7.7%), compared to 23 of 604 pregnancies that occurred after HIV diagnosis (3.8%) (p = 0.024).

Among the 81 WLHA who declared having had a pregnancy termination, 71.7% had had only one abortion, which was the same situation as 66.7% of 24 WNLHA. Among WLHA, 21.4% of abortions occurred in the first pregnancy, 21.4% in the second pregnancy, 15.9% in the third pregnancy, and 41.3% from the fourth pregnancy onwards, while in WNLHA, these percentages were 33.3%, 16.7%, 16.7%, and 33.3%, respectively (data not shown).

As to pregnancy planning, 65.7% of the pregnancies that occurred in WLHA were not planned and, of those, 9.7% were terminated by an induced abortion. Among WNLHA, 59.6% of the pregnancies were not planned, and 4.7% of them were terminated by an induced abortion (data not shown).

Regarding reasons to terminate a pregnancy (we could only retrieve the reason for termination in 127 of the 180 induced abortions), among pregnancies occurred in WNLHA (n = 32), 31.2% of pregnancies were terminated because the woman and/or her partner did not want a child at the time, 31.2% because the woman and/or her partner did not have the financial means to raise a child, 21.9% because the woman was not married and/or living with a partner or was in a relationship considered unfit for the birth of a child; 9.4% because her parents and/or her parents-in-law did not accept the pregnancy, and 6.3% for other reasons. Among pregnancies that occurred in WLHA before the HIV diagnosis (n = 74), the main reason to terminate a pregnancy was that she/her partner didn't want a child at that time (40.5%), followed by a relationship that was considered unfit for the birth of a child (21.6%), not to have financial conditions to raise a child (20,3%), not to have family or family-in-law support to have a child (13.5)% and other reasons (4.1%). On the other hand, among pregnancies occurred in WLHA after the HIV diagnosis (n = 21), the main reason to abort was that the woman was living with HIV/the fear of having a seropositive child (47.6%), followed by not to have the financial means to raise a child (19.1%), not to want a child at that time (14.3%), not to be living with a partner or to be in a relationship considered unfit for the birth of a child (9.5%) and other reasons (9.5%).

Regarding the description of characteristics related to the life course of women (Table 1), WLHA were characterized, at the time of the interview, as being older, having a lower education level, less family support, a greater number of sexual partners and a higher percentage of non-white skin, when compared to WNLHA (p<0.05). Regarding the specific context of each pregnancy (Table 2), WLHA were using more contraception when they got pregnant, had previously had more children, and were less often living with a partner, when compared to WNLHA (p<0.05).

Tables 3 and 4 show a description of the characteristics of WLHA and WNLHA, comparing women who had abortions to women who did not. WLHA who had abortions were older, had a higher education level and a greater number of sexual partners, when compared to the WLHA that declared having no abortion. WNLHA who declared having terminated a pregnancy also had a higher education level and a higher number of sexual partners, compared to those who had not had a pregnancy termination (Table 3). Regarding the characteristics linked to the specific context of each pregnancy, pregnancies occurring in WLHA were more frequently ended in an induced abortion if the women were older at the time of pregnancy, had previously had children, and were not living with a sexual partner at the time of the pregnancy. The only characteristic linked to the specific context of each pregnancy associated with an induced abortion in WLHA at the descriptive analysis was to be living with a sexual partner at the time of pregnancy (Table 4).

### Uni and Multivariable analysis

Having a higher education level and a greater number of sexual partners at the time of the interview and not living with a partner during the index pregnancy were associated with the declaration of an induced abortion in the univariable analysis in WLHA and WNLHA (Table 5). Among WLHA, being older at the time of the interview (between 40 and 49 years) and having had children before the index pregnancy were also associated with voluntary pregnancy termination. All variables that were significant in the univariable analysis remained statistically significant in the adjusted model for WLHA. Among WNLHA, only having a higher education level and a greater number of sexual partners were associated with the outcome in the multivariable model.

## Discussion

In the analysis of the reproductive decisions of women, it is possible to notice that twice as many pregnancies in WLHA resulted in an induced abortion when compared to pregnancies that occurred in WNLHA (6.5% versus 2.9%). This higher prevalence is especially true before the diagnosis, but remains higher even after the woman knows she is living with HIV.

There are few studies that examine the association between HIV and induced abortion [42], especially regarding the comparison between WLHA and WNLHA [13]–[15], [38]. Moreover, the scenario in which these studies were performed is different. Most of them took place in countries where abortion is safe and allowed by law. In addition, the way of measuring the outcome and the predictor was different. In this paper, the outcome was analyzed in each pregnancy. When the practice of abortion was analyzed, using woman as a sampling unit (instead of using pregnancy, as it is done in other studies), voluntary termination of pregnancy was still more frequent among WLHA than among WNLHA (13.0% versus 4.9%), which agrees with surveys conducted in Brazil and in Europe [15], [32].

The prevalence of abortion in both groups was expected to be lower than the prevalence reported in other studies that used women as sampling units [32], [34], [36]. In these studies, if a woman claims to have had a pregnancy termination, she is classified in the abortion group, no matter how many pregnancies she has had. The methodology used in the present study allows for the consideration of different outcomes for each pregnancy. Therefore, a pregnancy may result in abortion, regardless of the outcome of other pregnancies.

Regarding the scenario in which abortion happens, it must be considered that in Brazil induced abortion is, as a rule, prohibited by law, which favors its underreporting. Moreover, Osis et al. (1996) highlighted that it is not only the illegality that influences induced abortion underreporting, but also the *“[meaning unintended pregnancy and its interruption have on each woman's mind]”* (Osis et al., 1996, page 450) [43] and the importance of motherhood for the construction of women's identity in a given society. Likewise, it is important to consider that because of the illegality and social disapproval of induced abortion and because of the role that motherhood plays in the construction of women's identity, it is expected that the prevalence of abortion reported in this paper was underestimated. According to Rossier (2003), this underestimation can range between 5% and 60%, being lower in scenarios in which abortion is widely practiced, supported by law or socially acceptable [44]. However, there is no gold standard with which to compare the findings of our study. The care taken in the data collection, with the establishment of a trust bond between interviewers and interviewees, may have contributed to the decrease in abortion underreporting. Furthermore, one of the elements that could encourage underreporting of abortion is the fact that the interview was held in health services with maternal and child guidance. In Brazil, these health services have a position strongly opposed to the practice of abortion, which could justify its underreport. However, in both groups interviews were conducted in the same context, which leads us to believe that there would be no difference in underreporting between groups. Moreover, due to the cross-sectional design based on the declaration of past information, not only induced abortion declaration can be biased but other variables, such as number of sexual partners and contraceptive use. Indeed, there is no way to measure if this bias is greater in one group or in the other.

WLHA had more pregnancies that ended in induced abortions during their lifetime, and HIV has been declared as being the main reason for some post-diagnosis abortions. However, HIV is not the main reason for most of these post-diagnosis abortions. They occur mainly due to a combination of factors related to the relationship with the partner, with socioeconomic conditions and with lack of social support for a pregnancy. In fact, the diagnosis of HIV seems to be associated with a decrease in the prevalence of induced abortion. Thus, the decision to interrupt a pregnancy involves social, cultural and economic determinants, not assigned, in most cases, to a single factor. Agreeing with this finding, qualitative studies of women living with HIV indicate that motherhood can be used by these women as a strategy to cope with the stigma of the disease [45].

The factors associated with the occurrence of abortion did not differ greatly between WLHA and WNLHA. With respect to factors linked to the life course of women, in both groups, having a higher education level and a greater number of partners was associated with pregnancy termination. Regarding factors related to the specific context of each pregnancy, living with a partner and having had children before the index pregnancy were associated with induced abortion among WLHA. We believe that these determinants were not associated with the practice of abortion among WNLHA due to the small number of WNLHA who reported having had abortions during their lifetime, which implies a lack of statistical power to demonstrate significant differences between groups.

According to our findings, WLHA are in a context of greater social vulnerability compared to WNLHA, what has already been highlighted by Mann (1992) [46]. This author indicated that populations that are more marginalized, stigmatized and that suffer discrimination have greater vulnerability to HIV. Therefore, WLHA in our study had a lower education level, were more commonly non-white, and had less family support when compared to WNLHA. Furthermore, the most commonly alleged reason for the termination of pregnancy among WLHA was the instability of the relationship with the partner. This declaration, coupled with the greater number of sexual partners stated by these women, may be related to the social imperative of searching for a stable alliance, which, ultimately, would be responsible for providing greater social support for these women. Thus, even if the determinants of abortion are similar in WLHA and WNLHA, its rate is higher in WLHA, given the context of greater social vulnerability in which they live.

It is also important to note that only 21.4% of abortions reported by WLHA and 33.3% declared by WNLHA were performed in the first pregnancy, which may indicate that abortion has been used in these populations primarily as a contraceptive method, rather than a form of delay of the onset of the reproductive life. This becomes even more evident when we consider that 65.7% of pregnancies occurred in WLHA and 59.6% of the ones occurred in WNLHA were unplanned. This makes sense in a country like Brazil, where reproductive planning policies had a very late start [47]–[49], where the population has limited access to contraception (especially emergency contraception) [50], [51] and where abortion is, as a rule, still illegal.

## Conclusions

WLHA are more likely to have a history of pregnancies ended in an induced abortion, although factors associated with the practice of abortion do not differ among WLHA and women in the general population. This finding indicates that WLHA are in a context of greater social vulnerability when compared to WNLHA. This vulnerability exposes them to a higher risk of an occurrence of unwanted pregnancies, since these women have less family support to solve problems, have more unstable relationships and use contraception less frequently. Therefore, health services need to be aware of the issues related to sexuality and women, since having a history of abortion could indicate a greater chance of living with HIV.

Although contraception has been used in Brazil since the 1960s, there are information and access barriers that prevent its use by part of the population, as opposed to what happens in European countries, such as France, where there is a quite strong contraceptive norm and where access to contraception is widespread [52]. Thus, while in France abortion is used to correct an eventual contraceptive failure, in Brazil it has even been used as a contraceptive option, in a scenario in which family planning is weak.

In Brazil, AIDS has always been discussed as decoupled from reproductive health. There are two separate programs: the National Policy for Integral Attention to Women's Health (PNAISM) [53], which aims to promote the improvement of living conditions and health of Brazilian women and contribute to the reduction of morbidity and mortality that reaches this population; and the National STD/Aids Policy [54], which aims to reduce the incidence of HIV/Aids and to expand access to diagnosis, treatment and care. Therefore, the idea of integrating women's health through prevention and treatment of Aids is really recent, although, as attested by the results of this research, issues related to reproductive health are directly related to HIV, not only due to the transmission of the virus but and especially due to social vulnerability.
